# Numerical Simulation and Experimental Validation of Cutting Mechanism of Carbon Fiber-Reinforced Thermoplastic Composites

**DOI:** 10.3390/polym18040464

**Published:** 2026-02-12

**Authors:** Xingfeng Cao, Xiaozhong Wu, Xianming Meng, Sai Zhang, Tong Song, Pengfei Ren, Tao Li

**Affiliations:** 1 China Automotive Technology and Research Center Co., Ltd., Tianjin 300300, China; 2 Key Laboratory of Mechanism Theory and Equipment Design of Ministry of Education, Tianjin University, Tianjin 300072, China

**Keywords:** CFRTP, numerical simulation, machining, machining quality, surface integrity

## Abstract

Carbon fiber-reinforced thermoplastic composites (CFRTP) are widely used in automotive, aerospace, and other industries due to their lightweight, high specific strength, recyclability, and superior thermal properties. However, their non-homogeneity and anisotropy present challenging machining characteristics, often leading to damage that deteriorates component performance. It is imperative to conduct numerical simulation and experimental studies on CFRTP to systematically analyze the relationship between cutting mechanisms and the surface integrity of CFRTP. This study aimed to establish an innovative three-dimensional micro-scale cutting numerical model that integrates the differentiated constitutive behaviors and damage criteria of carbon fibers, matrices, and fiber–matrix interfaces—enabling precise characterization of micro-scale damage evolution during cutting. By combining simulation with experimental verification, it unveils the material removal mechanisms and processing damage causes of CF/PEEK, and further pioneers the quantification of the gradient correlation between fiber orientations (0°, 45°, 90°, and 135°) and fracture modes, cutting forces, and surface integrity, thereby addressing the gap of micro-mechanism and quantitative analysis in CFRTP machining. The micro-scale damage mechanisms revealed by the model directly reflect the intrinsic response of individual fibers in the tow, and the collective effect of these micro-behaviors determines the macro-scale machining performance observed in the experiments. A right-angle cutting experiment was conducted to validate the accuracy of the micro-scale numerical model. The mechanisms of fiber fracture, damage patterns, and chip morphology were systematically compared. The experimental results demonstrate good agreement with the outcomes of the numerical simulations. This study aims to bridge the gap between theoretical understanding and practical application of the cutting mechanisms in CFRTP, providing valuable insights for advancements in manufacturing processes.

## 1. Introduction

The increasing utilization of advanced composite materials, particularly CFRTP, has revolutionized industries such as aerospace, automotive, and manufacturing due to their exceptional mechanical properties, lightweight nature, and superior durability. CFRTP combine the high strength-to-weight ratio of carbon fibers with the impressive toughness and recyclability of thermoplastic matrices, making them a preferred choice for high-performance applications. However, the machining of CFRTP, specifically the cutting process, presents significant challenges due to the anisotropic and heterogeneous nature of these materials. Understanding the cutting mechanisms involved is essential for optimizing machining parameters, reducing tool wear, and enhancing overall product quality. The tool wear is one of the key factors affecting the machining quality of composites [1]. During the cutting process, the high hardness and abrasiveness of carbon fibers can cause rapid wear, blunting, and even chipping of the tool edge, which in turn exacerbates damages such as fiber tearing and interface debonding, and at the same time deteriorates the surface roughness and dimensional accuracy of the machined parts.

Koplev et al. [2] investigated chip formation and cutting forces in carbon/epoxy composites through orthogonal cutting experiments. It was revealed that chip formation in carbon/epoxy composites is dominated by brittle fracture, with cutting forces positively correlated to fiber orientation angle, providing a foundational reference for thermoset composite machining. Zhang et al. [3] partitioned the cutting zone of unidirectional fiber-reinforced composites into fracture, compression, and rebound regions, and developed a mechanical model to predict shear forces during orthogonal cutting. The study indicates that material removal mechanisms primarily depend on fiber orientation. The model effectively predicts shear force variation under different fiber orientations, but it simplifies the cutting zone as a homogeneous medium and ignores the discrete characteristics of fiber and matrix, leading to deviations in shear force prediction for composites with high fiber content.

The cutting strategy significantly influences machining quality. Wang et al. [4] investigated milling strategies (up and down milling) and cutting parameters’ impact on void defect formation in carbon fiber-reinforced polymers (CFRPs), especially with obtuse fiber-cutting angles. It was concluded that down milling with low cutting speed and high feed rate minimizes void defects, which is valuable for optimizing CFRP machining parameters. Their experiments underscore the strategy’s pivotal role in void formation. Down milling with low cutting speeds and high feed rates achieves fewer void defects and higher material removal rates. Wang et al. [5] examined void defect formation mechanisms on upper surfaces of unidirectional CFRP laminated disks across various fiber-cutting angles (0° to 180°) using circular orthogonal milling, attributing surface voids primarily to fiber delamination, bending-induced, and shear-induced fractures. The study comprehensively identifies three main sources of void defects, but it does not quantify the contribution of each mechanism to void formation, and the circular orthogonal milling method differs from conventional linear cutting, reducing the direct reference value for practical machining. Additionally, researchers have analyzed temperature rise during high-speed CFRP milling [6], studying how thermal conductivity varies with fiber orientation, which affects cutting temperature increases. Their results show that cutting temperature rises most significantly when fiber orientation is 90°, but the study only considered CFRP and did not compare temperature characteristics with thermoplastic composites, failing to address the impact of matrix thermal conductivity differences on cutting temperature. Zitoune et al. [7] conducted orthogonal cutting experiments and numerical simulations on carbon/epoxy composites, focusing on the influence of fiber orientation, tool geometry, and cutting depth on cutting forces and chip formation. They established a 2D numerical model based on fracture mechanics, using the virtual crack extension method to calculate the energy release rate, and confirmed that tool rake angle, relief angle, and cutting depth significantly affect cutting forces. However, this study only targeted 0° fiber orientation and thermoset composites, ignoring the differentiated damage mechanisms of thermoplastic matrices with high ductility, and the 2D model cannot capture out-of-plane damage such as delamination.

With the advancement of numerical simulation techniques, many researchers have employed finite element modeling to analyze CFRP processing. Su [8,9] investigated cutting forces and specific cutting energy characteristics of CFRP across various fiber orientations and cutting depths, using finite element models to study the impact of cutting speed on damage formation. The study found that specific cutting energy decreases with increasing cutting speed and increases with cutting depth, providing a basis for optimizing cutting parameters. Calzada et al. [10] proposed a 2D finite element model to depict material damage modes during CFRP chip formation, predicting machining forces and fiber characteristic lengths in chips. Phadnis et al. [11] developed a three-dimensional macroscopic finite element model for drilling composite laminates, considering complex tool-workpiece interface motion, and predicting drilling forces, torque, and damage with user-defined material models. They also established a finite element model for ultrasonic vibration-assisted drilling of CF/epoxy, validated through experimental verification [12]. The model accurately predicts drilling forces and torque, and confirms that ultrasonic vibration reduces drilling damage. Oliveira et al. [13] monitored CFRP milling via acoustic emission (AE) and infrared (IR) thermography, investigating the effects of tool condition, cutting speed, and feed direction on machining temperature, surface integrity, and AE signal features. Their findings showed that tool wear aggravates surface defects and increases roughness, with 45° feed direction causing the most severe damage, and AE signals being more sensitive to tool wear and parameter variations than cutting forces for online monitoring. Ameur et al. [14] constructed a finite element model for flax/epoxy composite drilling using the Hashin failure criterion and Puck criterion, realizing the prediction of thrust force and intra-ply damage, and verifying the influence of stacking sequence on machining quality. However, macroscopic finite element models do not capture fiber–matrix interrelations or damage evolution during processing. Therefore, many researchers have explored microsimulation of CFRP. Rao et al. [15,16] developed a 2D micro-cutting model for CFRP, analyzing fiber failure modes under different directions. The model reveals that fiber failure modes transition from bending fracture to shear fracture with increasing fiber orientation angle, advancing the understanding of micro-scale cutting mechanisms. Although effective in illustrating material removal mechanisms, this model lacks geometric feature information for intuitive analysis of matrix and fiber damage evolution or chip formation mechanisms. In addressing these limitations, Qi et al. [17] established a 3D multiphase orthogonal cutting model of CFRP, defining constitutive models for carbon fibers and matrix using user subroutines. Their simulation results provide intuitive insights into fiber–matrix damage evolution and fiber failure mechanisms, overcoming the limitations of 2D and macroscopic models. Their simulation results provide intuitive insights into fiber–matrix damage evolution and fiber failure mechanisms. Wang et al. [18] applied the equivalent homogenization method to construct a microscopic perspective finite element model of CFRP, uncovering material damage evolution and highlighting significant effects of fiber orientation on material removal mechanisms. The machinability of CFRP hinges primarily on fiber and matrix performance and their combined influence on chip formation mechanisms [19]. Presently, numerical simulations of cutting for fiber-reinforced composites predominantly concentrate on thermosetting materials.

Notably, Wang et al. [20] conducted a systematic comparative study on the cutting mechanisms of carbon/epoxy (thermoset) and CFRTP via experiments and finite element simulations. They developed differentiated material models: an orthotropic linear elastic constitutive model for carbon/epoxy composites, and a 3D elastic–plastic damage model incorporating 3D stress components for CFRTPs, with damage initiation and evolution determined by Hashin–Puck criteria and strain energy density. Through orthogonal cutting experiments under four fiber orientations (0°, 45°, 90°, and 135°), they verified that CFRTPs exhibit distinct cutting behaviors from thermoset composites, including shorter crack propagation, longer chip formation at 0° and 45° orientations, and suppressed subsurface damage at 135° orientation. They also reported that CFRTPs require higher cutting forces at 0°, 45°, and 135° orientations compared to carbon/epoxy composites. This study provides valuable insights into the differences between thermoset and thermoplastic composite cutting, but it focuses on macroscopic comparative analysis and does not delve into the microscale damage evolution of CFRTP components or the quantitative correlation between fiber orientation and fracture modes.

Hocheng and Puw [21] conducted a comparative analysis of chip formation during drilling processes for CFRP and carbon fiber/acrylonitrile butadiene styrene (CF/ABS). Their experimental results indicate that CF/ABS exhibits strong ductility and undergoes ABS plastic deformation, resulting in continuous long chips, whereas CFRP produces fragmented chips due to its relatively brittle epoxy resin matrix. This study pioneeringly compared the chip formation characteristics of thermoset and thermoplastic composites, but it only involved drilling processes and did not extend to orthogonal cutting, and the CF/ABS matrix differs from the Polyetheretherketone (PEEK) matrix in this study in terms of ductility and thermal stability, limiting the direct application of the conclusions. Their experimental results indicate that CF/ABS exhibits strong ductility and undergoes ABS plastic deformation, resulting in continuous long chips, whereas CFRP produces fragmented chips due to its relatively brittle epoxy resin matrix. Besides recyclability, CFRTP (CF/ABS) demonstrates higher ductility but poorer thermal conductivity compared to CFRP. The different properties of thermoplastic and thermosetting matrices significantly influence processing outcomes, leading to notable disparities in processing and damage behaviors among composite materials [22]. Xu et al. further confirmed that matrix properties are the primary factor causing differences in machining behavior between thermoset and thermoplastic composites, but the study only focused on turning processes and did not analyze the impact of matrix properties on cutting mechanisms. Therefore, detailed studies on the cutting mechanisms of CFRTP are essential.

Current macro homogenized models fail to capture microdamage such as fiber fracture and interface debonding; neglect matrix plasticity and temperature dependence, out-of-plane damage, and 3D stress; and lose discrete damage characteristics. To address these gaps, this study integrated simulation with experimental verification to unveil the material removal mechanisms and processing damage causes of CF/PEEK. A microscopic orthogonal cutting model was developed to simulate CF/PEEK at four typical fiber-cutting angles (0°, 45°, 90°, and 135°). The study examines fiber fracture and surface morphology formation mechanisms at different fiber angles during processing. Experimental validation of the simulation results confirms their accuracy, offering theoretical guidance for optimizing CFRTP processing quality. Quantitative correlations between fiber orientation and chip morphology, cutting force, and tool wear were established, and the gradient influence law of fiber angle on cutting force was clarified, providing refined theoretical support for the optimization of CFRTP processing parameters. The structure of the remaining part of this paper is arranged as follows: Section 2 introduces the numerical simulation method for the microscopic material removal mechanism; Section 3 presents the results of the cutting numerical simulation; Section 4 elaborates on the experimental device and scheme for orthogonal cutting; Section 5 compares and analyzes the simulation and experimental results and discusses the cutting mechanism; Section 6 summarizes the whole paper.

## 2. Numerical Simulation of Microscopic Material Removal Mechanisms

### 2.1. Establishment of a Micro-Scale Milling Simulation Model

**Figure 1:**
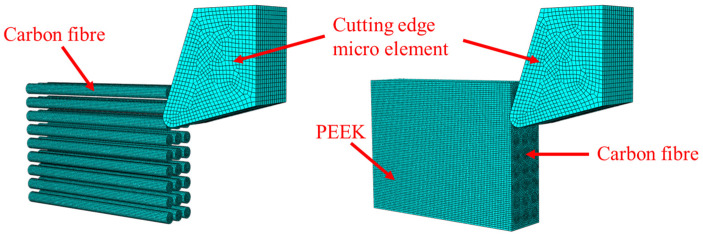
Microscopic orthogonal cutting model diagram (0°).

The microscopic orthogonal cutting model is shown in Figure 1. Due to the limitations of macro-scale milling models in accurately analyzing fiber damage mechanisms, a micro-element approach (orthogonal cutting) was employed to numerically simulate CF/PEEK at four typical fiber angles (0°, 45°, 90°, and 135°). The selection of the four typical fiber angles (0°, 45°, 90°, and 135°) is based on the existing literature [23,24]. These four fiber orientations (0° parallel, 45° oblique, 90° perpendicular, and 135° reverse oblique) encompass the critical relative orientations between fibers and the cutting direction, enabling the systematic characterization of the anisotropic cutting behavior of CFRTP. The simulation was conducted using Abaqus/CAE 2023 with associated subroutine versions compatible with Visual Studio 2019.

**Figure 2:**
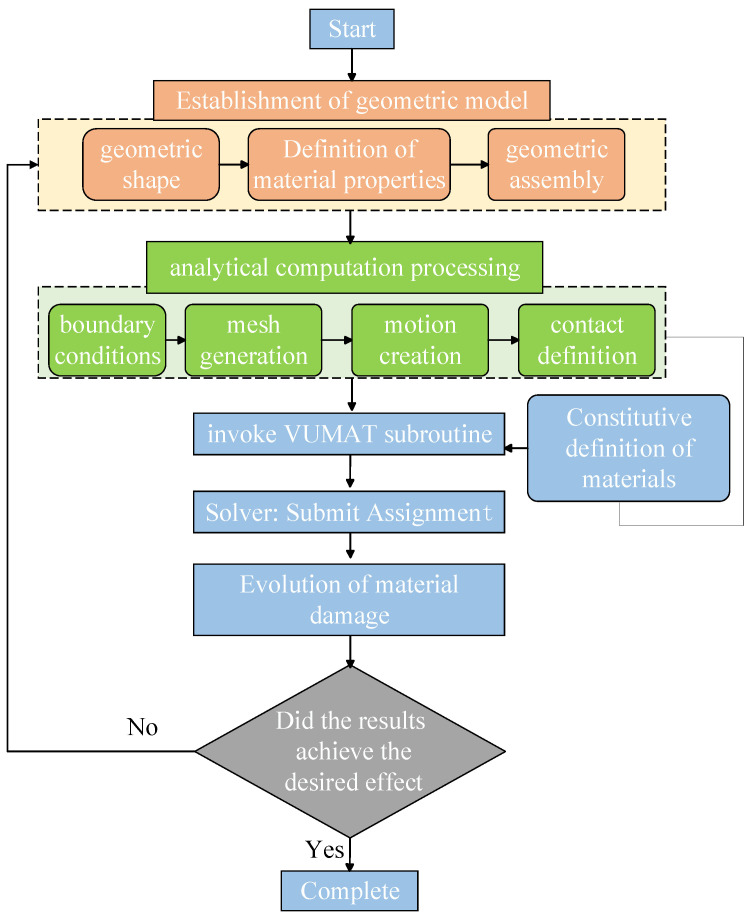
Flowchart of Abaqus numerical analysis.

Abaqus/CAE divides the analysis and computation process into several functional modules, including geometric model creation, analysis computation handling, and subroutine invocation. Geometric model creation entails developing models of the shapes for analysis, assigning relevant material properties, and assembling multiple models. Analysis computation handling is critical and varies in approach, significantly impacting results. It involves defining boundary conditions, contact methods, motion types, and mesh division. Subroutine invocation enhances the definition of material damage evolution for more precise numerical simulation results. The specific workflow of numerical simulation is illustrated in Figure 2.

### 2.2. Definition of Fiber-Cutting Angles

**Figure 3:**
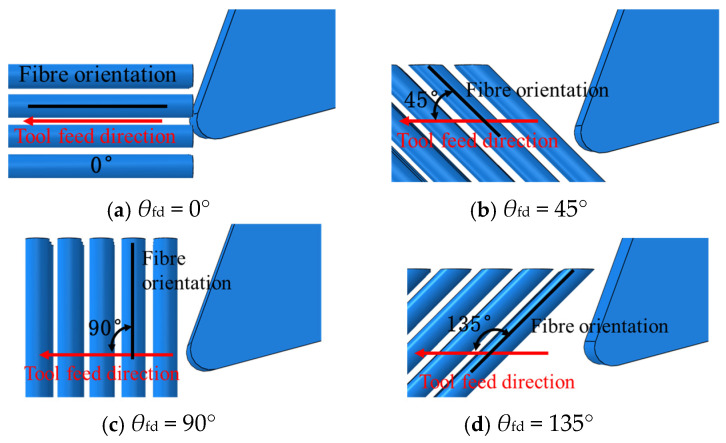
The definition of fiber orientation angle in the numerical simulation of cutting.

The definition of the fiber-cutting angle (*θ*_fd_) in orthogonal cutting refers to the clockwise angle between the cutting tool feed direction and the direction of the fibers, as illustrated in Figure 3.

### 2.3. Setting Conditions and Mesh Partitioning

**Figure 4:**
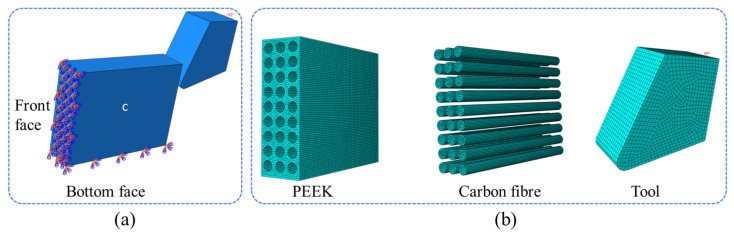
Numerical simulation diagram: (**a**) diagram illustrating the model constraints; (**b**) component mesh division.

After setting up the orthogonal cutting model in Abaqus, it is crucial to set boundary conditions, interactions, and loads for a more accurate computational analysis. The geometric model for orthogonal cutting simulation in the 0° fiber direction is depicted in Figure 4a. Following assembly, define the properties of the PEEK material in Abaqus to precisely analyze carbon fiber damage evolution, and call a subroutine when submitting the job.

Before submitting the job for analysis, computational processing needs to be conducted. Firstly, constrain the CF/PEEK model, as depicted in Figure 4a: fully constrain the bottom and front faces. The specific constraints are as follows:

(1) $$\left\{\begin{array}{l} U_{1}=U_{2}=U_{3}=0\\ U_{R1}=U_{R2}=U_{R3}=0 \end{array}\right.$$

The tool is set as a rigid body with a reference point (RP) established. The RP is coupled to the tool to control its motion, where the tool’s displacement speed is v. $V_{RX}$, $V_{RY}$, and $V_{RZ}$ are the cutting speeds in the X, Y, and Z directions, respectively. The constraints on the tool are as follows:

(2) $$\left\{\begin{array}{l} V_{X}=0;V_{Y}=V;V_{Z}=0\\ V_{RX}=0;V_{RY}=0;V_{RZ}=0 \end{array}\right.$$

Mesh division is shown in Figure 4b. It can be seen that the mesh density of the tool is lower than that of PEEK and carbon fiber. In the established model, the tool width is 28 μm. The specimen dimensions are 100 μm × 72 μm × 24 μm, and the fiber diameter is 7 μm. Because the tool is a rigid body, the requirements for mesh division are not high, and the form and size of the mesh do not affect the simulation results. For ease of calculation, a larger global size is used. For PEEK and carbon fiber, finer mesh division results in more accurate simulation calculations. Taking a fiber-cutting angle of 0° as an example, the finite element simulation adopts eight-node linear hexahedral elements, namely C3D8R elements, totaling 10,140 mesh elements. Both fiber and matrix use C3D8R mesh division with the neutral axis algorithm (minimizing mesh transitions). The element type is Explicit-linear-3D stress-reduced integration-element deletion, with 147,100 mesh elements for PEEK matrix division and 225,000 mesh elements for carbon fiber division. The unit size of PEEK is 0.01, and that of the tool is 0.05. Penalty contact methods are used to characterize interactions between surface contacts, establishing contacts between the tool and fiber, tool and PEEK, with a friction coefficient of 0.3 for the tool and specimen [25]. It can be confirmed that μ = 0.3 can minimize the deviation between simulation and experiment, by comparing the simulation results of different friction coefficients with the experimental cutting force and chip morphology. Additionally, adhesive contacts are established between the matrix and carbon fiber.

### 2.4. Constitutive Model and Damage Criteria

#### 2.4.1. Carbon Fiber

The carbon fiber was modeled as a linear elastic transversely isotropic material with failure defined by the maximum stress criterion. Carbon fiber is a typical linear elastic and transversely isotropic material [26,27]. Based on elasticity theory, the constitutive model of carbon fiber can be defined by Equation (3) [28]:

(3) σ=Cε

where *C* is the stiffness matrix, *σ* is the stress tensor, and *ε* is the strain tensor.

(4) $$C=\left(\begin{array}{cccccc} \frac{E_{11}\left(1-v_{23}v_{32}\right)}{1-B} & \frac{E_{11}\left(v_{21}+v_{31}v_{23}\right)}{1-B} & \frac{E_{11}\left(v_{31}-v_{21}v_{32}\right)}{1-B} & 0 & 0 & 0\\ \frac{E_{22}\left(v_{12}+v_{13}v_{32}\right)}{1-B} & \frac{E_{22}\left(1-v_{13}v_{31}\right)}{1-B} & \frac{E_{22}\left(v_{32}+v_{12}v_{31}\right)}{1-B} & 0 & 0 & 0\\ \frac{E_{33}\left(v_{13}+v_{12}v_{23}\right)}{1-B} & \frac{E_{33}\left(v_{23}+v_{13}v_{21}\right)}{1-B} & \frac{E_{33}\left(1-v_{12}v_{21}\right)}{1-B} & 0 & 0 & 0\\ 0 & 0 & 0 & G_{12} & 0 & 0\\ 0 & 0 & 0 & 0 & G_{23} & 0\\ 0 & 0 & 0 & 0 & 0 & G_{13} \end{array}\right)$$

Among them, 1, 2, and 3 represent, respectively, the longitudinal fiber and two perpendicular transverse ones; *E_ij_*, $\nu_{ij}$, and *G_ij_* (*i*, *j* = 1, 2, 3) represent, respectively, Young’s modulus, Poisson’s ratio, and shear modulus. $B=v_{12}v_{21}-v_{23}v_{32}-v_{13}v_{31}-2v_{21}v_{32}v_{13}$.

The failure of carbon fiber is defined by the maximum stress criterion [26], which means failure occurs when stress exceeds the ultimate strength of the material in that direction. Specifically, when any damage variable reaches 1, the fiber fails, and the corresponding elements delineated in the geometric model are deleted. The material constitutive model and damage criterion of carbon fiber are defined through the VUMAT subroutine. Refer to Equation (5) for details:

(5) $$\left\{\begin{array}{l} Longitudinal\;tensile\;failure\left(\sigma_{11}\geq 0\right)\;d_{t1}^{f}=\frac{\sigma_{11}}{X_{t}}\\ Transverse\;tensile\;failure\left(\sigma_{22}\geq 0\;\mathrm{or}\;\sigma_{33}\geq 0\right)\;d_{t2}^{f}=\frac{\sigma_{22}}{Y_{t}},d_{t3}^{f}=\frac{\sigma_{33}}{Y_{t}}\\ Longitudinal\;compressive\;failure\left(\sigma_{11}<0\right)\;d_{c1}^{f}=\left|\frac{\sigma_{11}}{X_{c}}\right|\\ Transverse\;compressive\;failure\left(\sigma_{22}<0\;\mathrm{or}\;\sigma_{33}<0\right)\;d_{c2}^{f}=\left|\frac{\sigma_{22}}{Y_{c}}\right|,d_{c3}^{f}=\left|\frac{\sigma_{33}}{Y_{c}}\right|\\ Internal\;shear\;failure\left(\sigma_{12}\neq 0\right)\;d_{s12}^{f}=\left|\frac{\sigma_{12}}{S_{12}}\right|\\ External\;shear\;failure\;d_{s23}^{f}=\max(\left|\frac{\sigma_{13}}{S_{13}}\right|,\left|\frac{\sigma_{23}}{S_{23}}\right|) \end{array}\right.$$

Among these, $d_{t1}^{f},d_{c1}^{f},d_{t2}^{f},d_{c2}^{f},d_{s12}^{f}$, and $d_{s23}^{f}$ represent damage variables for the failure of carbon fibers, with the superscript “*f*” indicating failure, and the subscripts “*t*”, “*c*”, and “*s*” representing tensile, compressive, and shear loads, respectively. The values “*X_t_*” and “*Y_t_*” denote longitudinal and transverse tensile strengths, respectively; *X*_c_ and *Y*_c_ represent longitudinal and transverse compressive strengths, *S*_12_, *S*_23_, and *S*_31_ indicate the shear strengths.

#### 2.4.2. Resin Matrix

The PEEK matrix was regarded as an isotropic elastoplastic material, and its plastic deformation and damage evolution were described by the Johnson-Cook (J-C) model. The PEEK matrix can be regarded as an isotropic elastoplastic material [29,30]. When stress is below the yield stress $\sigma_{y0}$, the material exhibits linear elastic behavior, which can be expressed by Equation (6).

(6) $$\sigma_{matrix}=E_{matrix}\varepsilon_{matrix}(\sigma_{matrix}<\sigma_{y0})$$

where $\sigma_{matrix}$, $\varepsilon_{matrix}$, and $E_{matrix}$, respectively, represent stress, elastic strain, and Young’s modulus of the PEEK matrix.

When the applied stress exceeds the yield strength of PEEK, the material enters a plastic state, and its plastic deformation can be defined using the Johnson–Cook (J-C) constitutive model [29,31]:

(7) $$\sigma \left(\varepsilon^{pl},{\dot{\varepsilon }}^{pl},T\right)=\left(A+B{\left({\bar{\varepsilon }}^{pl}\right)}^{n}\right)\left(1+Cln\frac{{\dot{\varepsilon }}^{pl}}{{\dot{\varepsilon }}_{ref}^{pl}}\right)\left[1-{\tilde{T}}^{m}\right]$$

where σ is the flow stress; *A* is the yield strength at the reference temperature and reference strain rate; *B*, *n*, and *C* are the strain-hardening coefficients, with strain-hardening power coefficient, and rate-hardening coefficient, respectively. $\varepsilon^{pl}$ is the equivalent plastic strain, ${\dot{\varepsilon }}^{pl}$ is the equivalent plastic strain rate, and ${\dot{\varepsilon }}_{ref}^{pl}$ is the reference strain rate. *T* is the temperature, *m* is the thermal softening power coefficient, *T* is the homologous temperature, and *T* is defined by Equation (8):

(8) $$\tilde{T}=\frac{T-T_{ref}}{T_{m}-T_{ref}}$$

where $T_{ref}$ is the room temperature, and $T_{m}$ is the melting temperature of PEEK.

Therefore, the initial J-C constitutive damage can be represented by Equation (9):

(9) $$\sigma_{s}=\left[d_{1}+d_{2}exp\left(-d_{3}\eta \right)\right]\left[1+d_{4}ln\left(\frac{{\dot{\varepsilon }}^{pl}}{{\dot{\varepsilon }}_{ref}^{pl}}\right)\right]\left(1+d_{5}\tilde{T}\right)$$

where $\sigma_{s}$ is the equivalent plastic strain of initial damage; *d*_1_, *d*_2_, *d*_3_, *d*_4_, and *d*_5_ are failure parameters; and η is the three-dimensional stress.

#### 2.4.3. Carbon Fiber–PEEK Matrix Interface

The bonding contact properties of the interface are characterized by the Cohesive criterion, and the mixed-mode damage evolution was defined using the B-K fracture criterion. As shown in Figure 4, the interface is the bonding region between carbon fibers and the PEEK matrix. The interactions inside the material to a certain extent determine the performance and behavior of the material, so the interface properties are crucial for the performance of the material and for predicting its behavior. The cohesive criterion is used to describe and analyze the adhesion of materials; therefore, the bonding contact properties of the interface are defined by the cohesive criterion:

(10) $${\left\{\frac{t_{n}}{t_{n}^{0}}\right\}}^{2}+{\left\{\frac{t_{s}}{t_{s}^{0}}\right\}}^{2}+{\left\{\frac{t_{t}}{t_{t}^{0}}\right\}}^{2}=1$$

where $t_{n}^{0}$, $t_{s}^{0}$, and $t_{t}^{0}$ represent the maximum strengths in the normal direction and two shear directions; $t_{n}$, $t_{s}$, and $t_{t}$ denote the traction stress in the normal direction and two shear directions.

The evolution of mixed-mode damage is defined through the B-K fracture criterion.

(11) $$G^{c}=G_{n}^{c}+\left(G_{s}^{c}-G_{n}^{c}\right){\left[\left(G_{s}+G_{t}\right)/\left(G_{n}+G_{s}+G_{t}\right)\right]}^{J}$$

where $G_{n}$, $G_{s}$ and $G_{t}$ are the energy release rates in the normal direction and two shear directions, and $G_{n}^{c}$, $G_{s}^{c}$ and $G_{t}^{c}$ are the critical energy release rates in the normal direction and two shear directions. J is the mixed damage parameter of the material.

J-C constitutive parameters: A (strain rate); B (strain-hardening coefficient); c (rate-hardening coefficient); m (thermal softening power exponent); n (strain-hardening power exponent). J-C damage parameters: $d_{1}$–$d_{5}$ (failure criterion parameters). The performance parameters of various parts of the CF/PEEK composite material used in the finite element simulation are listed in Table 1.

## 3. Cutting Numerical Simulation

**Figure 5:**
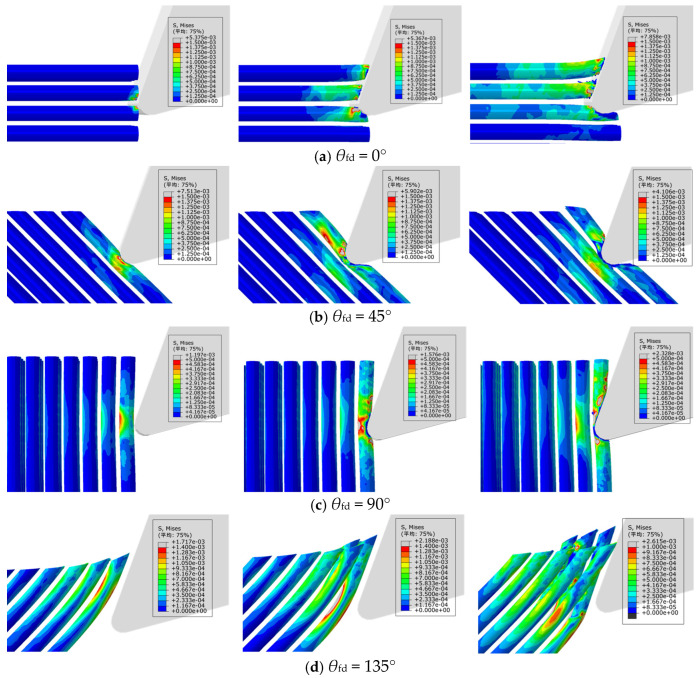
Numerical analysis diagram of fiber-cutting angles at: (**a**) 0°; (**b**) 45°; (**c**) 90°; (**d**) 135°. (Avg: 75%).

The fracture patterns exhibited by different fiber orientations vary significantly, affecting the surface quality after milling. To analyze the fiber fracture patterns more clearly and intuitively, the PEEK matrix was hidden in the post-processing of the numerical simulation. The numerical simulation results of the micro-material removal mechanisms are shown in Figure 5.

Figure 5 is a stress contour diagram, and its unit is GPa. Stress distribution exhibits obvious directional characteristics: at 0° and 135°, stress is concentrated in a narrow region along the cutting edge; at 45°, stress propagates obliquely along the fiber direction with a diffusion range; at 90°, stress is uniformly distributed across the fiber cross-section, showing a wider concentration zone. As shown in Figure 5a, when *θ*_fd_ = 0°, it can be observed that there is a significant stress concentration phenomenon in the fibers in contact with the cutting edge, and the fibers undergo bending deformation and delamination from the cutting edge of the front face. Therefore, for fibers with a cutting angle of 0°, the main failure mode of the fibers is bending fracture. As shown in Figure 5b, when *θ*_fd_ = 45°, the stress concentration mainly occurs in the fibers at the contact area of the cutting edge tip. After the fiber is compressed by the cutting edge tip, severe deformation occurs, and the stress is transmitted obliquely downward along the feed direction to the fibers behind, causing the fibers to be locally subjected to tension. Therefore, for fibers with a cutting angle of 45°, the main failure mode of the fibers is compressive crushing fracture.

As shown in Figure 5c, when *θ*_fd_ = 90°, the feed direction of the cutting edge is perpendicular to the fiber direction. The cutting edge compresses the resin matrix, transmitting stress to the fibers, thereby causing bending deformation in the fibers. The direction of stress transmission is along the direction of tool feed. In this case, the fibers are subjected to compressive fracture due to the compression by the cutting edge, achieving material removal. As shown in Figure 5d, it can be observed that there are differences in material removal between *θ*_fd_ = 135° and the other three fiber angles. The other three angles involve initial contact of the cutting edge tip with the fibers, whereas at *θ*_fd_ = 135°, the cutting edge of the front face makes initial contact with the material. As the cutting process continues, the front face exerts compression on the fibers, causing bending deformation in the fibers behind. Failure occurs when the stress exceeds the material’s ultimate strength. For a fiber-cutting angle of 135°, the main failure mode of the fibers is bending fracture.

## 4. Right-Angle Cutting Experiment

### 4.1. Experiment Materials and Setup

The experiment used thermoplastic carbon fiber-reinforced composite (CF/PEEK), provided by Jiangsu Junhua Special Engineering Plastics Co., Ltd. (Changzhou, China). The reinforcing phase consists of T700 carbon fibers with a fiber content of 66%. The laminate plate was stacked in the sequence [0]_16_ with a thickness of 2 mm. The laminate was fabricated via hot compress molding technology: the stacked prepreg layers were placed in a mold, heated to 380 °C, and maintained at a pressure of 6 MPa for 120 min. After the molding process, the laminate was cooled naturally to room temperature. Specimens were cut to dimensions of 50 mm × 20 mm × 2 mm using water jet cutting. Manufacturer-provided CF/PEEK performance parameters are detailed in Table 2.

**Figure 6:**
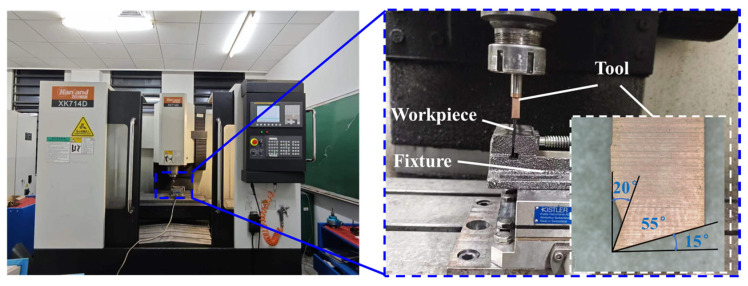
Right-angle cutting-experiment apparatus.

The right-angle cutting experiment was conducted on a CNC vertical milling machine (XK714D, Hanland, Hanzhong, China), as depicted in Figure 6. Specimens were secured using fixtures. The cutting tool was an uncoated hard alloy boring cutter with a shaft diameter of 10 mm, a cutting edge width of 7 mm, and front and back angles of 20° and 15° respectively, with a blade edge radius of 5 μm. The spindle rotation was locked during the cutting process.

### 4.2. Experimental Methodology

According to the relevant studies on CFRP machining [9,36], the parameters of the right-angle cutting experiment are shown in Table 3. The definition of fiber orientation is consistent with Section 2.2. After completing the experiment, the morphology of the cutting surface was observed using a cold field emission scanning electron microscope (Hitachi, Regulus 8100, Tokyo, Japan). The orthogonal cutting experiments on fiber-reinforced composites show that a cutting speed of 0.5 m/min can effectively avoid the change in material properties caused by excessive cutting temperature, and at the same time ensure that phenomena such as fiber fracture and chip formation are clearly observable [16]. In addition, the cutting tool employed in the experiments featured a rake angle of 20°, a clearance angle of 15°, and an edge radius of 5 μm.

## 5. Results and Discussion

### 5.1. Analysis of Cutting Force

**Figure 7:**
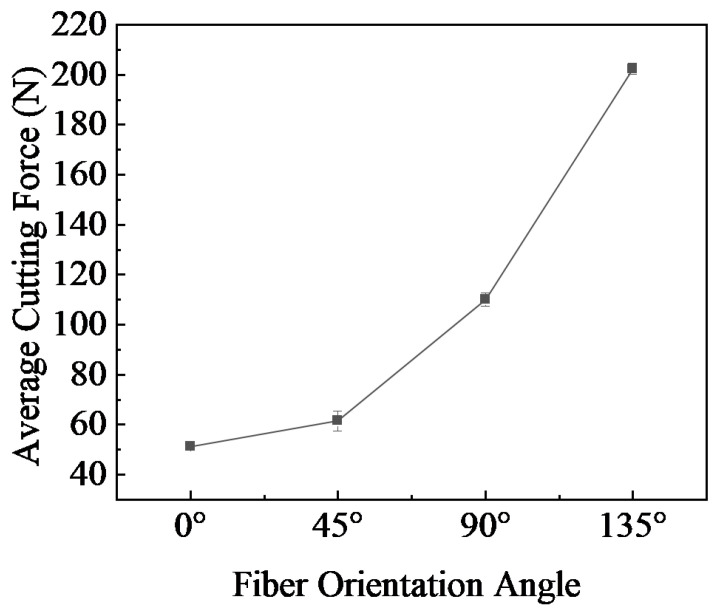
The average cutting force at different fiber angles.

Figure 7 focuses on the variation in cutting forces of CF/PEEK at four fiber orientation angles. The cutting force shows a gradient increasing characteristic. From 0° to 45°, the cutting force increases slowly from 51.40 N to 61.50 N. Because the fibers are parallel to the cutting direction, stable shearing is dominant, and the resistance increases gently. From 45° to 90°, the cutting force rises to 109.94 N, as the fibers turn perpendicular to the cutting direction, and the force becomes extrusion and shearing, leading to a rapid increase in load. From 90° to 135°, the cutting force rises to 202.40 N. The fibers form an obtuse angle with the cutting direction, with pulling-breaking as the dominant mode; coupled with subsurface damage and fiber fracture, the resistance attains its peak.

**Figure 8:**
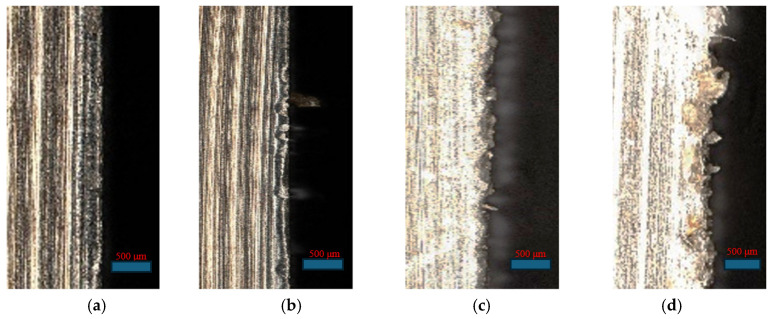
SEM image of cutting edge: (**a**) 0° fiber-cutting angle; (**b**) 45° fiber-cutting angle; (**c**) 90° fiber-cutting angle; (**d**) 135° fiber-cutting angle.

The degree of tool wear is closely related to the magnitude of the cutting force. There are significant differences in the cutting-edge morphologies at different fiber-cutting angles in Figure 8, which intuitively reflects the influence of the cutting angle on the machining quality: the cutting edge integrity is the best at 0°. As the angle increases, the wear, material adhesion, and damage degree of the cutting edge gradually intensify, reaching the most severe state at 135°. The cutting edge in Figure 8a has excellent integrity, a smooth surface, extremely little fiber adhesion, and no obvious wear or microcracks. This phenomenon is due to the uniform stress distribution and gentle cutting process during 0° cutting, which ultimately results in regularly curled chips. The cutting edge surface in Figure 8b exhibits slight roughness, accompanied by mild serrated wear and local fiber–matrix adhesion, which is attributed to the combined effects of local stress concentration induced by oblique cutting and friction during fiber extrusion. The cutting edge in Figure 8c is bent and deformed. Delamination, debonding residues, and early micro-crack initiation can be observed. This is caused by the transverse bending thrust on the fibers when the tool is in vertical contact with the fibers. The cutting edge wear in Figure 8d is the most severe, with a large amount of blocky fiber–matrix adhesion, which is attributed to the repeated impact and intense friction caused by the random fracture of fibers during 135° cutting.

### 5.2. Material Removal Mechanism

**Figure 9:**
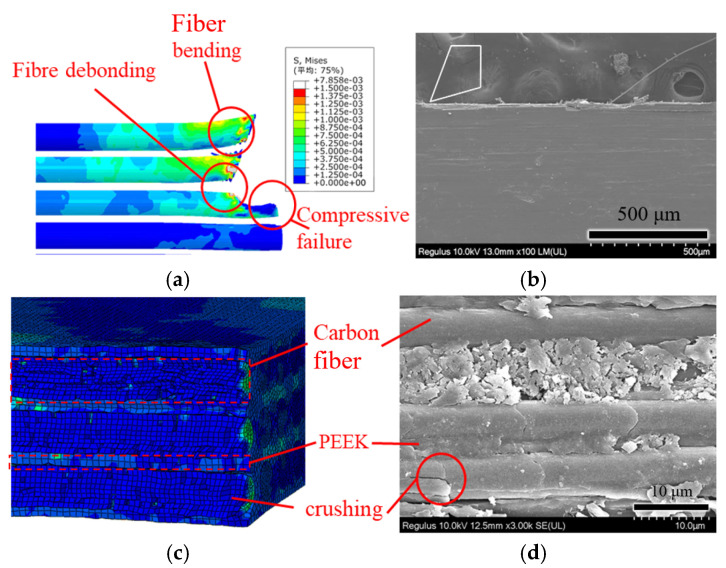
Simulation and experimental comparison of fiber-cutting angles at 0° cutting angle: (**a**) simulation cutting diagram; (**b**) experimental cutting diagram; (**c**) simulation cutting morphology; and (**d**) experimental cutting morphology. (Avg: 75%).

Different cutting depths have a notable impact on the machining surface quality of PEEK. However, the purpose of this paper is to explore the cutting-related mechanisms and machining characteristics under four fiber angles. Moreover, the model established in this paper is a micro-scale model, and to minimize computational load, the influence of cutting depth on material mechanisms and chip formation is not considered herein. To validate the accuracy of the numerical simulation, a comparative analysis was conducted between the simulation results and experimental findings. Figure 9 illustrates the comparison between simulation and orthogonal cutting experiments at *θ*_fd_ = 0°. It can be observed that the primary material removal mechanism in fiber materials is bending fracture. Fibers above the cutting edge are peeled off under the action of the rake face, leading to fiber pullout. Meanwhile, fibers below the cutting edge experience compressive forces from the tool, resulting in fiber crushing. As shown in Figure 9d, fiber crushing and fiber exposure phenomena are visible on the cutting surface, consistent with the numerical simulation results (see Figure 9c).

**Figure 10:**
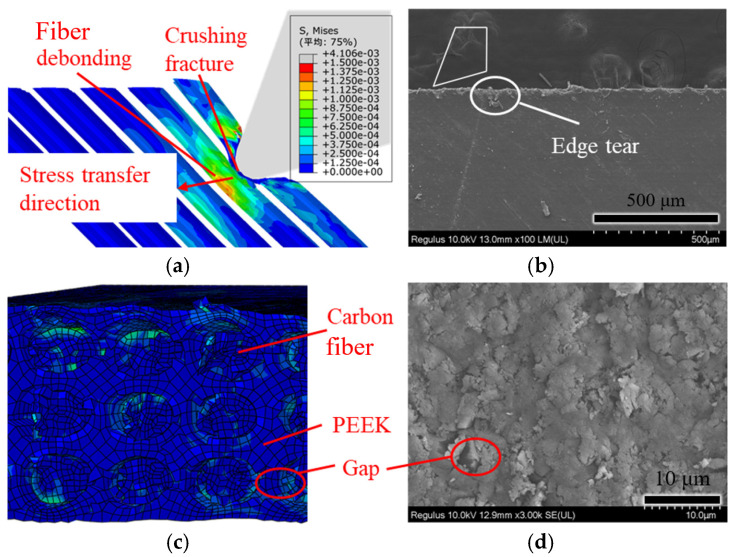
Simulation and experimental comparison of fiber-cutting angle at 45°: (**a**) simulation cutting diagram; (**b**) experimental cutting diagram; (**c**) simulation cutting morphology; and (**d**) experimental cutting morphology. (Avg: 75%).

Figure 10 shows the simulation and experimental comparison of cutting at a fiber angle of 45°. From Figure 10a, it can be seen that stress is transmitted obliquely downward along the cutting edge in the feed direction, causing tear damage along the fiber direction. As shown in Figure 10b, edge-tearing damage can be observed along the fiber direction. The morphology of simulation cutting in Figure 10c shows that the broken fibers are not in the same plane as the matrix, and there are gaps between the fibers and the PEEK matrix, consistent with the morphology of orthogonal cutting in Figure 10d. This may be because, as the tool advances, stress is transmitted obliquely downward, leading to sub-surface fiber bending deformation, which can cause debonding between the fibers and the matrix, thus forming gaps.

**Figure 11:**
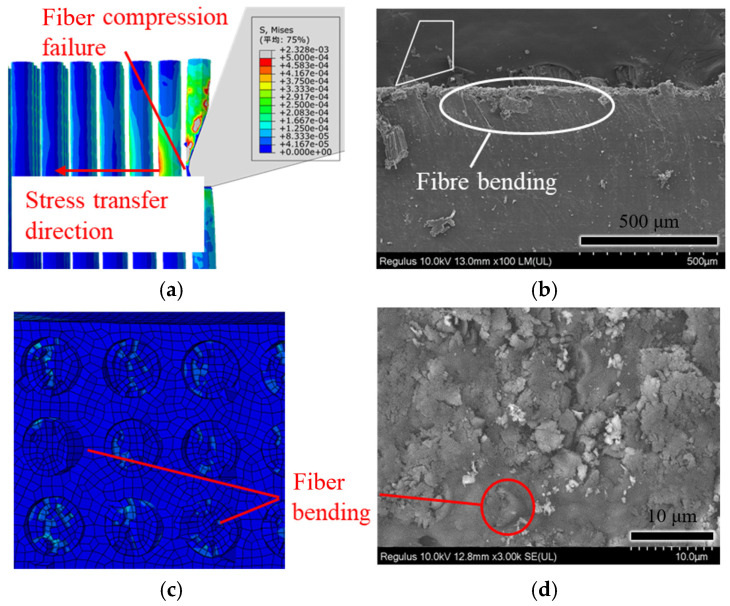
Simulation and experimental comparison of fiber-cutting angle at 90°: (**a**) simulation cutting diagram; (**b**) experimental cutting diagram; (**c**) simulation cutting morphology; and (**d**) experimental cutting morphology. (Avg: 75%).

Translation: Figure 11 shows the simulation and experimental comparison of cutting at a fiber angle of 90°. From Figure 11a, it can be observed that the primary mode of fiber fracture is a compression fracture. Under the squeezing action of the cutting edge, stress concentrates at the cutting tip and propagates along the tool’s feed direction. Additionally, under the squeezing action of the tool, fibers beneath the cutting surface undergo bending deformation, as shown in Figure 11b. From Figure 11c, it can be observed that the fracture surface of the fibers bends towards the cutting direction, consistent with the observations in Figure 11d.

**Figure 12:**
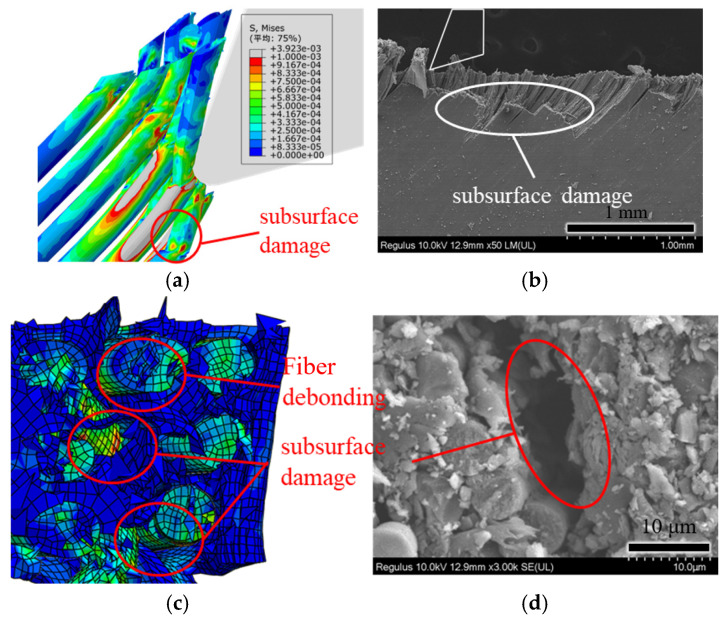
Simulation and experimental comparison of fiber-cutting angle at 135°: (**a**) simulation cutting diagram; (**b**) experimental cutting diagram; (**c**) simulation cutting morphology; and (**d**) experimental cutting morphology. (Avg: 75%).

Figure 12 illustrates the comparison between simulation results and experimental results of orthogonal cutting at a fiber-cutting angle of 135°. At this angle, fiber fracture primarily occurs through bending. Due to the initial contact of the cutting edge with the material, the leading cutting edge exerts compressive forces on the material during the cutting process. Under the compressive action of the tool, the fibers undergo significant deformation, leading to fracture when the strain exceeds their maximum load-bearing capacity. From Figure 12a, it can be observed that fibers fracture in the subsurface, resulting in severe subsurface damage (see Figure 12b). As shown in Figure 12c,d, the surface integrity of the machined surface at 135° is comparatively poorer than at the other three fiber-cutting angles. Post-machining morphology reveals significant fiber–matrix debonding and irregular fiber alignment. Furthermore, subsurface damage is pronounced. At an angle of 135°, fiber fracture occurs through bending fracture, which is the core mechanical reason for the most severe damage [37]. As the fibers first come into contact with the rake face of the tool, they break under the extrusion of the rake face. As the tool feeds, the tool tip contacts the fibers, causing the fibers below the cutting surface to break. The stress exerted by the front-row fibers on the back-row fibers exceeds the ultimate strength of the fibers, leading to the fracture and failure of the back-row fibers.

### 5.3. Chip Analysis

The section provides a comparative analysis of chip formation through simulation and orthogonal cutting, elucidating the mechanisms of material removal. Experimental results indicate that in CF/PEEK machining, chip morphology is notably influenced by fiber angle, closely tied to fiber fracture modes.

**Figure 13:**
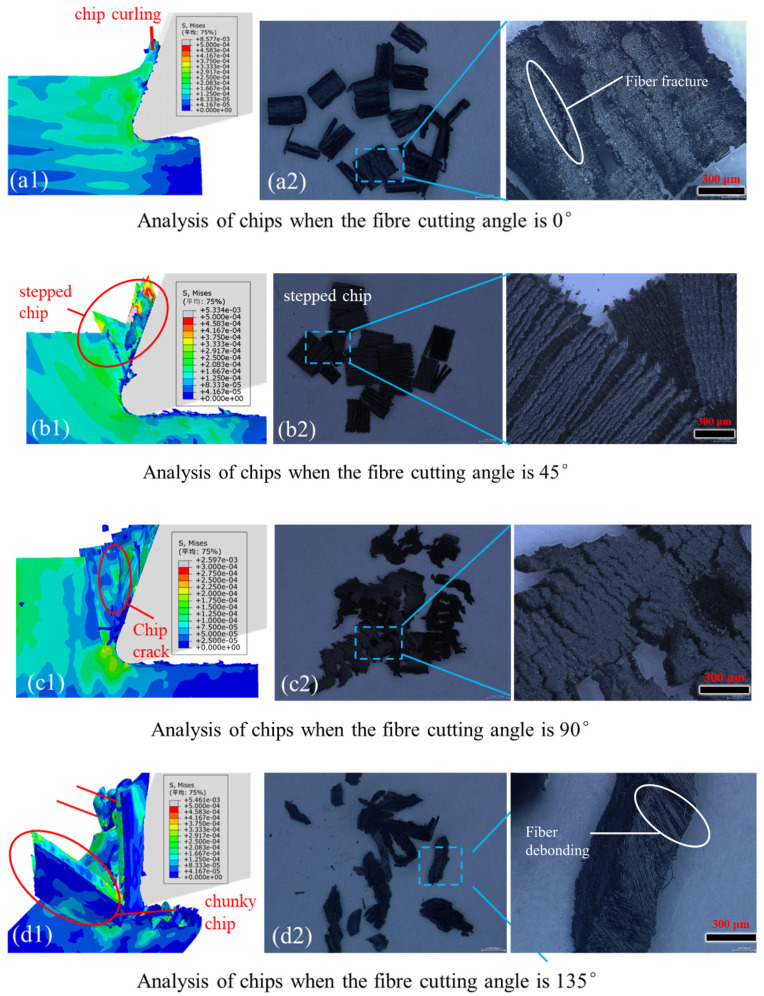
Chip Analysis Diagrams for Fiber-cutting Angles: (**a1**) simulation results of 0°; (**a2**) experimental results of 0°; (**b1**) simulation results of 45°; (**b2**) experimental results of 45°; (**c1**) simulation results of 90°; (**c2**) experimental results of 90°; (**d1**) simulation results of 135°; (**d2**) experimental results of 135°. (Avg: 75%).

When the fiber-cutting angle is 0°, as observed from Figure 13(a1), the leading cutting edge exerts upward and forward thrust on the chips, causing them to bend. Additionally, the bending fracture of fibers contributes to the curved deformation of chips. From the chip morphology in Figure 13(a2), it is evident that the surface integrity of the chips is good, attributed to the excellent ductility of the PEEK matrix and strong fiber adhesion. Moreover, fractured fibers can be observed on the chip surface, corroborating the conclusion that fiber bending fracture has occurred.

As shown in Figure 13(b1), when the fiber-cutting angle is 45°, the chips are in a stair-step shape, and the broken fibers are progressively squeezed out under the cutting edge thrust. From Figure 13(b2), it can be observed that the chip surface exhibits strong stratification. Under high magnification, the chip surface appears as a stair-step structure. Numerical analysis results are consistent with experimental results.

From Figure 13(c1), it can be seen that there are cracks in the chip edge that are aligned with the fiber direction. The voids observed in Figure 13(c2) that are also aligned with the fiber direction further indicate the phenomenon of fiber–matrix debonding during cutting. Additionally, it can be noticed that the integrity of the chip edge is poor. This may be due to the fact that the tool feed direction is perpendicular to the fiber direction, causing stress to concentrate in the area where the fiber contacts the cutting edge. As a result, fibers located at the edge experience more severe deformation due to a lack of sufficient external support, leading to debonding and deteriorating the integrity of the chip edge.

As shown in Figure 13(d1), when the fiber-cutting angle is 135°, the chips exhibit significantly different characteristics in morphology and integrity compared to the three fiber angles mentioned above: the chips are blocky. Additionally, severe debonding between fibers and the matrix can be observed. From Figure 13(d2), it can be seen that the chips are irregular and have poor integrity, further confirming the significant deformation and subsequent debonding of fibers due to compressive forces at the 135° cutting angle. Furthermore, due to the compressive action of the cutting edge, the orientation of fibers may appear irregular. This irregular arrangement reflects the random deformation and fracture modes of fibers under compressive stress, which significantly differ from the arrangement and fracture patterns of fibers at other cutting angles. This phenomenon illustrates the complexity of the stress state of fibers and the material removal process at a 135° cutting angle, thereby affecting the morphology and quality of the chips. The fiber cutting angle (θ) directly determines the stress state and fracture mode of carbon fibers, while differences in fracture modes further affect the formation and evacuation efficiency of chips, with the specific correlation rules shown in Table 4.

The surface characteristics of the chips are jointly determined by the fiber fracture mode, fiber–matrix interaction, and cutting stress state. At 0°, the fibers exhibit a bending fracture. The high ductility of the PEEK matrix makes the chip surface smooth and intact, with only a small number of broken fiber ends exposed and no obvious debonding. At 45°, it is an extrusion crushing fracture, the surface shows a stepped delamination, accompanied by fiber–matrix separation and extrusion scratches. At 90°, it is a compression fracture; the integrity of the chip edge is poor, with micro-cracks and voids in the fiber orientation distributed, and the surface continuity is damaged. At 135°, although it is a bending fracture, due to the dominance of tangential stress, the surface is the roughest and most irregular, presenting a blocky structure, with severe fiber–matrix debonding and disordered arrangement. The surface quality of the chips is directly related to the machining performance: the surface is the best at 0°, corresponding to stable cutting and low tool wear; the surface is the worst at 135°, reflecting severe stress effects and serious damage, which provides a key basis for optimizing cutting parameters and selecting fiber orientation.

## 6. Conclusions

This paper establishes a mesoscopic model of milling simulation using Abaqus (right-angle cutting) and defines the constitutive behaviors of CF/PEEK components. Numerical simulations were conducted for four typical fiber-cutting angles (0°, 45°, 90°, and 135°). Additionally, an experimental platform for right-angle cutting was constructed to validate the corresponding fiber-cutting angles experimentally. By comparing the experimental results with the simulation results, the accuracy of the finite element simulation was verified. The removal mechanisms and damage of materials were compared, and an analysis of chip morphology was conducted, laying a theoretical foundation for milling experiments, such as material removal mechanisms and surface morphology. The conclusions drawn are as follows.

Compared with the 2D-macroscopic models focusing on CFRPs and the macroscopic comparison of CFRTPs, the 3D microscopic model in this study accurately captures its micro-damage, clarifies the laws of differential fracture and cutting force gradient, fills the quantitative gap in the cutting mechanism of thermoplastic composites, and is superior to the single-dimensional analysis of previous studies. An analysis of CF/PEEK materials at four typical fiber-cutting angles using finite element simulation was conducted, validated against experimental results of right-angle cutting. The fracture modes for the 0°, 45°, 90°, and 135° fiber-cutting angles, respectively, are: bending fracture, crushing fracture, compressive fracture, and bending fracture.

The cutting force shows a gradient increasing trend with the fiber angle. This is mainly because, at this angle, the fibers are mainly broken by pulling and accompanied by severe subsurface damage. From the side views of the right-angle cutting, it was observed that the 0° fiber-cutting angle had the least damage, while the 135° angle showed the most severe damage. At 90°, fibers were notably bent in the direction of the tool feed, and at 45°, there was minor tearing damage.

Surface morphology analysis post-cutting revealed that at the 0° fiber-cutting angle, there were crushed fibers and exposed fibers; at 45°, there were voids; at 90°, there were bent fibers visible on the surface; and at 135°, there was significant subsurface damage. There is a positive correlation between tool wear and cutting force. The cutting edge has the best integrity and the least wear at 0°. The cutting edge wears most severely at 135°, accompanied by a large amount of fiber–matrix adhesion. Because fibers first contact the tool’s rake face and break under extrusion, as the tool feeds, the tool tip fractures subsurface fibers, and the stress from front-row fibers on back-row ones exceeds their ultimate strength, leading to the latter’s failure.

In future work, we will discuss the impact of different cutting depths on machining quality, and the influence of different friction coefficients on material removal should be further discussed and studied. These research results provide guidance for the selection of optimal fiber orientation and cutting parameters in the processing of CFRTPs, which helps to reduce subsurface damage and improve surface integrity.

## Figures and Tables

**Table 1:** Performance parameters of different parts of CF/PEEK composite materials used in numerical analysis [25,32,33,34,35].

Material	Property	Parameter Value
Carbon fiber	elastic modulus	*E*_11_ = 235 GPa, *E*_22_ = 14 GPa, *E*_33_ = 14 GPa
	Poisson’s ratio	*v*_12_ = *v*_13_ = 0.2, *v*_23_ = 0.25
	Sheer strength	*S* = 0.38 GPa
	shear modulus	*G*_12_ = 28 GPa, *G*_13_ = 28 GPa, *G*_23_ = 5.5 GPa
	longitudinal strength	*X*_t_ = 4.9 GPa, *X*_c_ = 2.7 GPa
	transverse strength	*Y*_t_ = 1.5 GPa, *Y*_c_ = 2.55 GPa
PEEK	elastic modulus	*E* = 4.1 GPa
	Poisson’s ratio	*v* = 0.38
	J-C constitutive parameters	*A* = 132 MPa, *B* = 10 Mpa, *C* = 0.034, *m* = 0.7, *n* = 1.2
	J-C damage parameters	*d*_1_ = 0.05, *d*_2_ = 1.2, *d*_3_ = 0.254, *d*_4_ = −0.009, *d*_5_ = 1
Interface	normal strength	$t_{n}^{0}$ = 43 MPa
	shear strength	$t_{s}^{0}$ = $t_{t}^{0}$ = 50 MPa
	fracture toughness	$G_{n}^{C}=1.7\;kJ/m^{2},G_{s}^{C}=2.0\;kJ/m^{2}$
	B-K index	*J* = 1.09

**Table 2:** The unidirectional CF/PEEK performance.

Property Parameters	CF/PEEK
Fiber type	T700
Fiber content	66%
Glass transition temperature	143 °C
Density	1.59 g/cm^3^

**Table 3:** Orthogonal cutting experimental parameters.

Fiber Angle (*θ*_fd_)	Cutting Depth	Cutting Speed
0°, 45°, 90°, and 135°	0.1 mm	0.5 m/min

**Table 4:** Correlation table of fiber angle, fracture mode, and chip morphology.

Fiber Angle	Fracture Mode	Characteristics of Chip Morphology
0°	Bending Fracture	Good integrity; curled; smooth chips from PEEK matrix’s ductility; fiber adhesion
45°	Crushing Fracture	Step-like; strong layering; fiber–matrix separation; visible layered structure on chip surface
90°	Compressive Fracture	Poor edge integrity; distinct fiber–matrix separation
135°	Bending Fracture	Block-shaped; severe fiber separation; disordered arrangement

## Data Availability

The original contributions presented in this study are included in the article. Further inquiries can be directed to the corresponding authors.
